# 

**Published:** 1903-06

**Authors:** 


Vol. XVIII. JUNE, 1903. No. 12.

### TEXAS Medical Journal.

Subscription, $1.00 a Year, in Advance.

Single Copies, 10 Cents.

PUBLISHED AT AUSTIN, TEXAS, BY

F. E. DANIEL, M. D.,

Proprietor.

Entered at the Postoffice at Austin, Texas, as Second-class Mail Matter.

